# Rice Soluble Starch Synthase I: Allelic Variation, Expression, Function, and Interaction With *Waxy*

**DOI:** 10.3389/fpls.2018.01591

**Published:** 2018-11-13

**Authors:** Qianfeng Li, Xinyan Liu, Changquan Zhang, Li Jiang, Meiyan Jiang, Min Zhong, Xiaolei Fan, Minghong Gu, Qiaoquan Liu

**Affiliations:** ^1^Key Laboratory of Plant Functional Genomics of the Ministry of Education/Key Laboratory of Crop Genetics and Physiology of Jiangsu Province/Jiangsu Key Laboratory of Crop Genomics and Molecular Breeding, College of Agriculture, Yangzhou University, Yangzhou, China; ^2^Co-Innovation Center for Modern Production Technology of Grain Crops of Jiangsu Province, Joint International Research Laboratory of Agriculture & Agri-Product Safety of the Ministry of Education, Yangzhou University, Yangzhou, China

**Keywords:** SSSI, allelic variation, soluble starch synthase, *waxy*, physiochemical properties, eating and cooking quality, rice (*Oryza sativa* L.)

## Abstract

Starch, which is composed of amylose and amylopectin, is the key determinant of rice quality. Amylose is regulated by the *Waxy* (*Wx*) gene, whereas amylopectin is coordinated by various enzymes including eight soluble starch synthases (SSSs), of which SSSI accounts for ∼70% of the total SSS activity in cereal endosperm. Although great progress has been made in understanding *SSSI* gene expression and function, allelic variation and its effects on gene expression, rice physicochemical properties and qualities, and interactions with the *Wx* gene remain unclear. Herein, *SSSI* nucleotide polymorphisms were analyzed in 165 rice varieties using five distinct molecular markers, three of which reside in an *SSSI* promoter and might account for a higher expression of the *SSSI^i^* allele in *indica* ssp. than of the *SSSI^j^* allele in *japonica* ssp. The results of *SSSI* promoter-*Beta-Glucuronidase* (β-GUS) analysis were consistent with the expression results. Moreover, analysis of near isogenic lines (NILs) in the Nipponbare (Nip) background showed that Nip (*SSSI^i^*) and Nip (*SSSI^j^*) differed in their thermal properties, gel consistency (GC), and granule crystal structure. Knockdown of *SSSI* expression using the *SSSI*-RNA interference (RNAi) construct in both *japonica* and *indica* backgrounds caused consistent changes in most tested physicochemical characteristics except GC. Moreover, taste value analysis (TVA) showed that introduction of the *SSSI* allele in *indica* or knockdown of *SSSI* expression in *japonica* cultivars significantly reduced the comprehensive taste value, which was consistent with the superior taste of *japonica* against *indica*. Furthermore, to test the potential interaction between *SSSI* and different *Wx* alleles, three NILs within the *Wx* locus were generated in the *indica* cv. Longtefu (LTF) background, which were designated as LTF (*Wx^a^*), LTF (*Wx^b^*), and LTF (*wx*). The *SSSI*-RNAi construct was also introduced into these three NILs, and physiochemical analysis confirmed that the knockdown of *SSSI* significantly increased the rice apparent amylose content (AAC) only in the *Wx^a^* and *Wx^b^* background and caused different changes in GC in the NILs. Therefore, the effect of *SSSI* variation on rice quality also depends on its crosstalk with other factors, especially the *Wx* gene. These findings provide fundamental knowledge for future breeding of rice with premium eating and cooking qualities.

## Introduction

Rice (*Oryza sativa* L.) is the most important carbohydrate and energy source for human nutrition. Rice yield has been greatly increased in recent decades (Sasaki et al., 2002; Zuo and Li, 2014), but improvements in grain quality remain limited and insufficient to meet the requirements of both the rice breeders and the consumers (Anacleto et al., 2015). Starch is the major component of rice, accounting for ∼90% of milled rice endosperm. Starch composition and structure are closely correlated with rice eating and cooking qualities (ECQs), especially taste properties of cooked rice (Tian et al., 2009). Although great efforts have been made to improve rice quality, and a number of ECQ-related genes have been identified and characterized (Aluko et al., 2004; Jeon et al., 2010; Phing et al., 2016), the underlying genetic mechanisms remain ambiguous as grain quality traits are complex.

Starch, which is the main determinant of rice ECQs, consists of two glucose homopolymers, amylose and amylopectin. Amylose is a linear polymer composed of 1,4-linked-α-D-glucan chains, whereas amylopectin is a highly branched glucan with α-1,6-glucosidic bonds that connect linear chains. Amylopectin is the major component of the total starch weight, and it contributes greatly to its semicrystalline nature. In rice endosperm, starch biosynthesis requires the cooperation of at least five groups of enzymes, namely adenosine diphosphoglucose (ADP) pyrophosphorylase (AGPase), granule-bound starch synthase (GBSS), soluble starch synthase (SSS), starch branching enzyme (SBE), and starch debranching enzyme (DBE) (Myers et al., 2000; Nakamura, 2002; Jeon et al., 2010). Amylose is mainly synthesized by GBSSI, which is encoded by the *Waxy* (*Wx*) gene. Therefore, differences in the apparent amylose content (AAC) in rice varieties are closely correlated with natural allelic variation at the *Wx* locus. The major *Wx* alleles are *Wx^a^, Wx^b^*, and *wx*, corresponding to high, intermediate, and zero AACs, respectively (Wang et al., 1995; Hirano et al., 1998; Zhang et al., 2017). Recently, a number of novel, natural *Wx* alleles have been reported, including *Wx^mp^, Wx^mq^*, and *Wx^op^* (Sato et al., 2002; Liu et al., 2009; Tian et al., 2009; Yang et al., 2013).

Amylopectin biosynthesis requires the coordination of a few key enzymes, including members of the SSS family, which contains the largest number of isoforms and is divided into four subfamilies based on the similarity of amino acid sequence. In rice, there are three genes encoding SSSII, two encoding SSSIII and SSSIV, but only one encoding SSSI. The single SSSI isoform accounts for ∼70% of the total SSS activity in cereal endosperm. Considering the fact that the single SSSI isoform provides a major contribution to amylopectin biosynthesis, experiments were carried out to mine microsatellites or single nucleotide polymorphisms (SNPs) in *SSSI* to facilitate later the breeding of high quality rice. Thus, three classes of microsatellites, designated as *SSS*-A, *SSS*-B, and *SSS*-C, were identified in 56 accessions of waxy rice varieties (Bao et al., 2002). In addition to the above three microsatellites, one more microsatellite, *SSS*-D, was found in 172 accessions of non-waxy rice varieties (Bao et al., 2006). Furthermore, by amplification of pooled deoxyribonucleic acid (DNA) and deeply parallel sequencing, a total of 73 SNPs and 8 insertions and deletions (Indels) were detected in the exons and introns of *SSSI* in 233 rice genotypes. Neither SNP nor Indel was functional (Kharabian-Masouleh et al., 2011). Expression assay indicated that *SSSI* has a constitutive expression pattern in all tested tissues of rice, including germinating seeds, roots, leaves, leaf sheaths, and panicles (Hirose and Terao, 2004). During the seed development process of rice, *SSSI* has high expression at the earliest phase of seed formation [1–3 days after flowering (DAF)], peaking expression at 5 DAF, and an almost constant expression throughout the late-milking stage of seed development (Ohdan et al., 2005). *In vitro* and *in vivo* data indicate that SSSI plays a distinct role in the synthesis of short amylopectin chains; *SSSI* mutants and related transgenic plants revealed that SSSI mainly synthesizes polymer chains with a degree of polymerization (DP) of 8–12 from the A chain (DP 6–7) and the external segments of B1 chains that emerge from the nearest branch point of amylopectin (Fujita et al., 2006; McMaugh et al., 2014; Wang et al., 2017). In addition, SSSI belongs to carbohydrate active enzyme (CAZy) glucosyltransferase family 5 (GT5) and possesses two conserved domains (GT5 and GT1) (Schwarte et al., 2013). The crystal structure of SSSI from barley was also solved (Cuesta-Seijo et al., 2013). Although knockout of *SSSI* alone causes no notable defects in rice seeds and starch granules, the complete loss of SSSI and SSSIIIa combined with reduced SSSIIa activity leads to rice sterility, but the presence of minimal SSSI activity is sufficient to reverse the sterile phenotype (Fujita et al., 2006; Hayashi et al., 2018). This indicates an important role for SSSI in seed development, and the function of SSSI is at least partially redundant with other SSS enzymes. Nevertheless, as the largest component of total soluble SS activity, whether SSSI interacts with Wx, the major contributor to rice amylose content, remains unknown.

In general, although great progress has been made in revealing the allelic variation, expression, and function of *SSSI* in amylopectin biosynthesis and seed development, studies focusing on the exploration and application of *SSSI* in rice-breeding programs are scarce. Similar to the allelic variation in the *Wx* gene, mining the natural functional variation in the *SSSI* gene could prove useful for breeding programs. Since allelic variation in *Wx* contributes greatly to differences in ECQs between *japonica* and *indica* (Jeon et al., 2010; Liu et al., 2014; Zhang et al., 2017), it is important to investigate more on the allelic diversity of *SSSI*, which is potentially conserved between the two subspecies, and explore its influence on rice quality. Moreover, it is also essential to explore the potential interactions between *SSSI* and different *Wx* alleles.

In this study, in order to investigate the allelic variation of *SSSI* as well as evaluate its influence on rice quality and potential interactions with the *Wx* gene, we conducted the following studies using a series of transgenic rice plants and NILs: (1) allelic variation of *SSSI* was mined from 165 rice varieties; (2) *SSSI* promoters of different lengths, mimicking the *japonica* and *indica* *SSSI* promoters, were cloned and characterized in transgenic rice; (3) the effects of downregulating the expression of *SSSI* on rice physicochemical properties were investigated using RNAi in both *japonica* and *indica* genetic backgrounds; (4) the effects of *indica*-type *SSSI* alleles were assessed and compared with that of the *japonica* background using the NILs; and (5) potential genetic interactions between *SSSI* and *Wx* were analyzed by suppressing *SSSI* expression in different NILs in *Wx*.

## Materials and Methods

### Plant Materials

Rice samples for allelic variation analysis included 106 *japonica*, 42 *indica*, 6 *javanica*, and 10 intermediate genotypes as well as 1 *O. rufipogon*. Two rice cultivars, LTF and Nip, which carry the *SSSI^i^* and *SSSI^j^* alleles, respectively, together with their *SSSI* suppression lines and NILs were used to investigate the expression profiles of the *SSSI* gene. To minimize the effects of different genetic backgrounds in the endosperm, *SSSI*-NIL was generated by introducing *indica* *SSSI^i^* into the Nip (*SSSI^j^*) genetic background, resulting in Nip-NIL-*SSSI^i^*. Nip-NIL-*SSSI^i^* and Nip-*SSSI^j^* were then used to study the influence of allelic variation of *SSSI* on rice quality. In addition, three typical *indica* rice varieties, Zhenshan97B (ZS97B), Teqin (TQ), and LTF and three typical *japonica* rice varieties, Wuxiang9915 (WX9915), Wuxiangjing9 (WXJ9), and Guanglingxiangnuo (GLXN), were used to investigate the expression pattern of *SSSI* in different subspecies. All rice plants were grown at the same experimental farm at Yangzhou University under identical climate conditions. Three replicate plots were used for all experiments, and the plots were arranged in a randomized block pattern. According to the experimental requirements, seed samples were collected at different developmental stages, at 5, 10, 15, and 20 DAF, and all other samples, including leaves, leaf sheaths, stems, and roots, were harvested during grain filling. All collected samples were immediately frozen in liquid nitrogen (LIN) and stored at -80°C.

### Sequence Diversity and Marker Analysis of *SSSI* in Two Rice Subspecies

Available sequences of the *SSSI* gene from genome databases as well as the *SSSI* sequences from 10 other rice varieties that were sequenced by us were used to identify homologous regions of two model rice species, namely *indica* 9311 and *japonica* Nip. To clarify sequence diversity at the *SSSI* loci, DNA sequences ranging from the *SSSI* promoter to the 3′-untranslated regions (UTRs), approximating 11.5 kb, were selected and aligned for 12 rice varieties. The Indels were used for developing sequence-tagged site (STS) markers, whereas SNPs, which included some restriction recognition sites, were useful for developing cleaved-amplified polymorphic sequence (CAPS) markers. Primers were designed using primer 5.0 software based on the diversity of the sequence (Supplementary Table 1).

### Vector Construction and Rice Transformation

Based on the available rice genome sequences, pairs of primers were designed to isolate differential lengths, 0.76 and 2.15 kb, of the 5′-flanking sequence of *OsSSSI*, mimicking *indica* and *japonica* *SSSI* promoters, respectively. The correct promoter sequences of *OsSSSI* were introduced upstream of the *GUS* reporter gene in the binary vector *pCAMBIA1300*. The fused expression vector (*pSSSI::GUS*) was then introduced into agrobacterium strain EHA105 and transformed into *japonica* Wuxiangjing9 (WX9) via agrobacterium-mediated transformation (AMT). The RNAi vector was constructed using plasmid *p1200*, in which a maize *ubiquitin* promoter controls the expression of an inserted DNA fragment that included sense-and antisense-specific coding regions of *SSSI*. This construct was introduced into *japonica* rice cv. Nip and *indica* rice cv. LTF to downregulate the expression of *SSSI* by AMTs in the two rice varieties.

### GUS Activity Assay of *pSSSI::GUS* Transgenic Rice

The GUS activity assay was performed as described previously (Li et al., 2018). In brief, a fluorometric assay was conducted by grinding various samples of tissues into a powder with LIN in a mortar and total proteins were extracted in a GUS extraction buffer. Reaction buffer containing 1 mmol L^-1^ 4-methyl-umbelliferyl-β-d-glucuronide was then used for the determination of GUS activity.

### Expression Analysis by Quantitative Real-Time PCR (qRT-PCR)

The total RNA was extracted from the collected tissues and followed by the DNaseI (QIAGEN, Hilden, Germany) treatment. Then, the RNA was purified using the RNeasy Plant Mini kit (QIAGEN) according to the manufacturer’s instructions. The ratios of the A260/A280 RNA samples were between 1.8 and 2.0, which were used for further analyses. An 1 μg sample of RNA was used for reverse transcription with the SuperScript first-strand synthesis system (Invitrogen, Carlsbad, CA, United States) according to the manufacturer’s instructions. Real-time PCR was performed using an ABI PRISMTM 7700 sequence detector system (Applied Biosystems, Carlsbad, CA, United States). The *Actin* gene was used as an internal control for normalization of gene expression (Li et al., 2010). Sequences of primers used to amplify genes in real-time-PCR (qRT-PCR) assays are listed in Supplementary Table 1.

### Flour Preparation and Physicochemical Analyses

Grain physicochemical properties of two typical rice cultivars, NIP of *japonica* rice variety and LTF of *indica* rice variety, and their transgenic lines, together with the NIL Nip-NIL-*SSSI^i^* were analyzed. The collected mature seeds were first dehusked and then polished with a grain polisher (Kett, Tokyo, Japan). The polished rice samples were ground into flour particles with a diameter less than 0.15 mm. The AAC of rice was then determined using the iodine colorimetric method. Gel consistency (GC) and Rapid Visco Analyzer (RVA) profiles were obtained as described previously (Li et al., 2018), and differential scanning calorimetry ((DSC) 200 F3; Netzsch Instruments: NA LLC, Burlington, MA, United States) was used to evaluate starch gelatinization and retrogradation temperature as previously described (Zhu et al., 2012). All tests were performed in triplicate.

### Taste Value and Cooking Properties

To determine the cooking properties of rice, 30 g of milled rice was washed in a stainless steel mesh container and transferred into an aluminum box with 40 mL distilled water. Rice was cooked in an electric rice cooker for 30 min. After 20 min of equilibration and 90 min of cooling at room temperature, the sensory properties of cooked rice were evaluated with an STA1B rice sensory analyzer (Satake, Japan).

### X-Ray Powder Diffraction

The crystalline structure of starch was analyzed by X-ray powder diffraction (XRD) using an X-ray diffractometer (D8, Bruker, Germany), and the relative crystallinity of starch was measured as previously described (Man et al., 2013).

### Attenuated Total Reflectance-Fourier Transform Infrared (ATR-FTIR) Spectroscopy

The structure of the external surface of native starch was analyzed by attenuated total reflectance Fourier transform infrared (ATR-FTIR) spectroscopy – using a Varian 7000 instrument – with a deuterated triglycine sulfate (DTGS) detector (PIKE Technologies, United States). Before deconvolution, spectra were corrected by a baseline in the region from 1200 to 800 cm^-1^. Absorbance values of starch at 1045 and 1022 cm^-1^ were extracted from the spectra after water subtraction, baseline correction, and deconvolution.

### Starch Molecular Weight Distribution

The molecular weight (MW) distribution of both native and debranched starch sample was determined by gel permeation chromatography (GPC) (PLGPC 220, Polymer Laboratories Varian, Inc., Amherst, MA, United States). Purified rice starch samples were debranched by isoamylase (EC3.2.1.68, EISAMY, Megazyme) with some minor modifications, as previously described (Zhu et al., 2012). In general, 10 mg of each starch sample was added to a 12 mL glass vial containing 5 mL of 0.01 mol/L acetate buffer (pH 4.2) and a micro stir bar. The glass vial was placed in a boiling water bath for 1 h, the solution was cooled to room temperature, isoamylase was added, and the vial was placed in a 40°C water bath for 36 h and then transferred to a boiling water bath for another 10 min. After cooling to room temperature, the sample was dried in a freeze-drier. For GPC analysis, 4 mg of freeze-dried native starch or debranched starch was dissolved in 4 mL dimethyl sulfoxide (DMSO) and incubated in boiling water for 24 h with constant stirring. Before injection of samples, starch solution was filtered through a 2.0 μm filter. The collected GPC data were transformed through integral equations based on dextrans of known MW, and transformed data were used to plot MW distribution curves. Using dextran standards, GPC data were reported as dextran-equivalent MW.

## Results

### Allelic Variation of *SSSI* in Different Rice Cultivars

**FIGURE 1:**
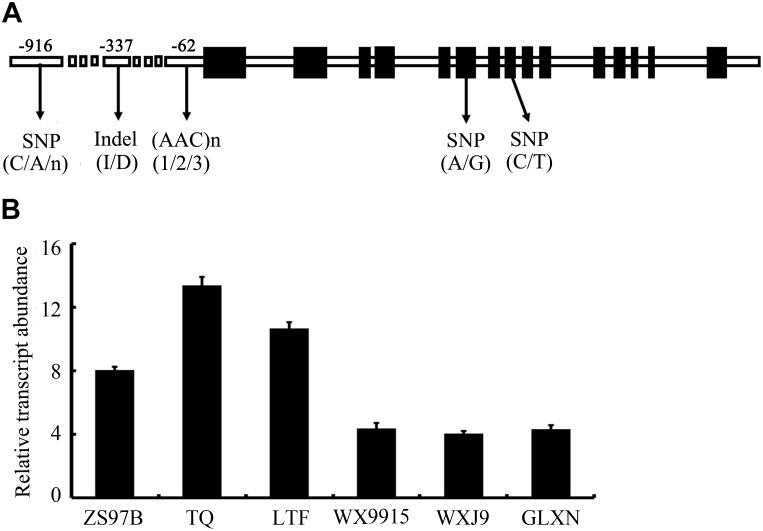
Schematic map of allelic variation of *Soluble Starch Synthases I* (*SSSI*) **(A)** and expression of *SSSI* in different rice varieties **(B)**. Total RNA was extracted from caryopses at 10 DAF and analyzed. A housekeeping gene *Actin1* was used as an internal control.

Polymorphism of the *SSSI* gene was identified by the alignment of *SSSI* sequences from 12 rice cultivars for which genomes are available online or were determined by us. Based on polymorphism data, four simple PCR-based distinct molecular markers were established, two in the promoter region and the other two in the exons (Figure 1A). In detail, there is an Indel in the promoter region and an STS marker S001 at -916 bp in the *SSSI* promoter, which was developed to identify this Indel. Moreover, a C/A SNP at -337 bp in the *SSSI* promoter was also identified and could be recognized by *Hae*II in *japonica* cultivars but not in *indica* cultivars. Similarly, in exon 6, there is an A/G SNP, which is closely correlated to an *Msp*I recognition site that is only present in the *japonica* cultivar. Furthermore, the PCR-*Mbo*I marker was developed in exon 8. Thus, one Indel marker and three newly established CAPS markers (PCR-*Mbo*I, PCR-*Hae*II, and PCR-*Msp*I) as well as the previously reported Simple Sequence Repeat (SSR) marker 488/489 (Bao et al., 2002) at -62 bp were used to distinguish different *SSSI* haplotypes.

To enhance our knowledge of allelic variation of *SSSI* and its impact on rice quality, it is crucial to analyze germplasm that spans a full range of phenotypic variation. Therefore, the five molecular markers were applied to the detection of numerous, diverse rice varieties, and eight haplotypes (I/A/C/C/3, I/A/C/C/2, I/A/C/C/1, I/A/C/A/2, I/A/C/A/3, D/G/T/n/1, D/G/T/n/2, and D/G/T/n/3) were distinguished from 165 varieties (Table 1). The four characters and one number representing each haplotype indicated the type of polymorphism at -916 bp in the *SSSI* promoter Indel, an A/G SNP in exon 6, a C/T SNP in exon 8, a C/A SNP or without PCR product (n) at -337 bp in the promoter, and the type of ACC microsatellite at -62 bp in *SSSI* promoter, respectively. Based on the results of Indel marker screening, these rice varieties were classified into two subgroups (Class-I and Class-II). Moreover, four newly developed markers were closely associated with each other in response to natural variation in the *SSSI* gene. More interestingly, the STS marker in the promoter and two of the CAPS markers in the coding region were completely linked. In other words, three mutation sites (I/A/C or D/G/T) coexist in the promoter and coding regions of *SSSI*. To sum up, 105 of the 106 *japonica* accessions have the I/A/C haplotype. For the 42 *indica* accessions, two haplotypes, I/A/C and D/G/T, correspond to 24 and 18 cultivars, respectively. Another CAPS marker located upstream of the transcription initiation codon was partly associated with the above three markers, representing a fourth polymorphic site in addition to the three *SSSI* haplotypes (I/A/C/C, I/A/C/A, and D/G/T/n). Moreover, 101 of the 106 *japonica* accessions have the I/A/C/C haplotype, and five *javanica* accessions also possess this allele. Meanwhile, the I/A/C/A allele was found in some other cultivars, including 4 *japonica* accessions, 11 *indica* accessions, and 6 intermediate genotypes. Surprisingly, the D/G/T/n allele was detected mainly in accessions. Regarding the microsatellite marker, three allelic classes of *SSSI* were identified in the examined samples, among which 123 accessions belonged to the type 3 allele, implying that it is the most common allele and is consistent with the results of the previous study (Bao et al., 2006). Therefore, the distribution of *SSSI* allele types is subspecies specific. Our results suggest that I/A/C/C/3 is the dominant allele type in *japonica*, hence it was named *SSSI^j^*, whereas the D/G/T/n/1 allele was named *SSSI^i^* because it is specific to *indica*.

**Table 1:** Allelic variation of the *SSSI* gene in 165 rice germplasms.

Gene type			Number	*Japonica*			*Indica*			*Javanica*	Intermediate	*O. rufipogon*
Group	Subclass	Allele		Non-waxy	Waxy	Total	Non-waxy	Waxy	Total	(Waxy)	(Waxy)	(Waxy)
	I-1	I/A/C/C/3	115	6	91	97	4	6	10	5	3	
	I-2	I/A/C/C/2	4	3		3	1		1			
Class-1	I-3	I/A/C/C/l	3		1	1	2		2			
	I-4	I/A/C/A/2	15	1	2	3	4	3	7		5	
	I-5	I/A/C/A/3	7		1	1	4		4		1	1
	II-1	D/G/T/n/1	16				3	11	14	1	1	
Class-2	II–2	D/G/T/n/2	4		1	1	1	2	3			
	II–3	D/G/T/n/3	1				1		1			
Total			165	10	96	106	20	22	42	6	10	1

*In the Allele lane, in total four characters and one number were used to represent each haplotype. In more detail, the first character I/D indicates the insertion or deletion event at -760 bp in the *SSSI* promoter; the second character A/G stands for the A/G SNP in exon 6 of *SSSI*; the third character C/T means the C/T SNP in exon 8; the fourth character C/A or n means the C/A SNP or without PCR product (n) at -337 bp in *SSSI* promoter; the last number 1/2/3 represents 11/9/8 copies of ACC microsatellite at -62 bp in *SSSI* promoter*.

### Comparison of *SSSI^j^* and *SSSI^i^* Expression Profiles

**FIGURE 2:**
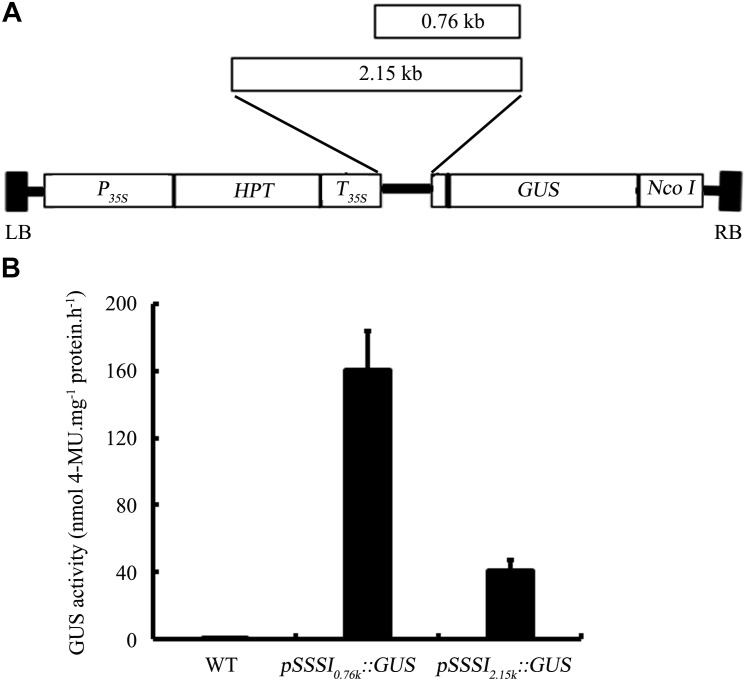
T-DNA structure of the *pSSSI::GUS* construct **(A)** and GUS activity in the endosperm of *pSSSI::GUS* transgenic rice **(B)**. WT indicates the untransformed wild-type plant. The seed samples analyzed here were collected at 10 DAF. Error bars represent the standard error.

First, we monitored the expression pattern of *SSSI* in various tissues of Nip, including seeds at different developmental stages. The result showed that *SSSI* could express in all the tested tissues, with the highest expression in the developing seeds at 15 DAF (Supplementary Figure 1). Next, we examined the expression of *SSSI* in *japonica* and *indica* cultivars to investigate the expression of *SSSI^j^* and *SSSI^i^* alleles and developing seeds from six representative rice cultivars, namely *indica* cv. ZS97B, *indica* cv. TQ, and *indica* cv. LTF and *japonica* cv. WX9915, *japonica* cv. WXJ9, and *japonica* cv. GLXN, which were selected for the analysis. The result of qRT-PCR showed that the overall transcript abundance of *SSSI* in *indica* was much higher than that in *japonica* (Figure 1B). To further confirm the potential transcriptional variation between *SSSI^j^* and *SSSI^i^*, different lengths of *SSSI* promoters (0.76 and 2.15 kb) were cloned to mimic the *indica* and *japonica* *SSSI* promoters (Figure 2A), and these were separately fused with the *GUS* reporter gene and transformed into rice to generate *pSSSI_0.76k_::GUS* from the *indica SSSI promoter* and *pSSSI_2.15k_::GUS* from the *japonica* *SSSI* promoters. After several rounds of selection, a number of homozygous transgenic lines from the two cultivars were obtained. After initial GUS activity assessment, one representative line from each transgenic event was selected for the comparison of transcriptional activity. The GUS activity results indicated that regardless of length, the *SSSI* promoter could drive the *GUS* reporter gene to express in all tested tissues, including anthers, embryos, endosperms, stems, stem nodes, seed hulls, leaves, and leaf sheaths, with the highest expression in stems and stem nodes (Supplementary Figure 2). Interestingly, the overall GUS activity was much higher in the *pSSSI_0.76k_::GUS* transgenic plants than in *pSSSI_2.15k_::GUS* rice plants. For example, the GUS activity in the endosperm of *pSSSI_0.76k_::GUS* transgenic plants was about three times higher than that in *pSSSI_2.15k_::GUS* rice plants (Figure 2B). The GUS activity result agrees with the above qRT-PCR result (Figure 1B), suggesting that *SSSI* transcription is higher in *indica* than in *japonica*.

### Suppression of *SSSI* Expression by RNAi and Its Effects on Rice Physicochemical Properties

**FIGURE 3:**
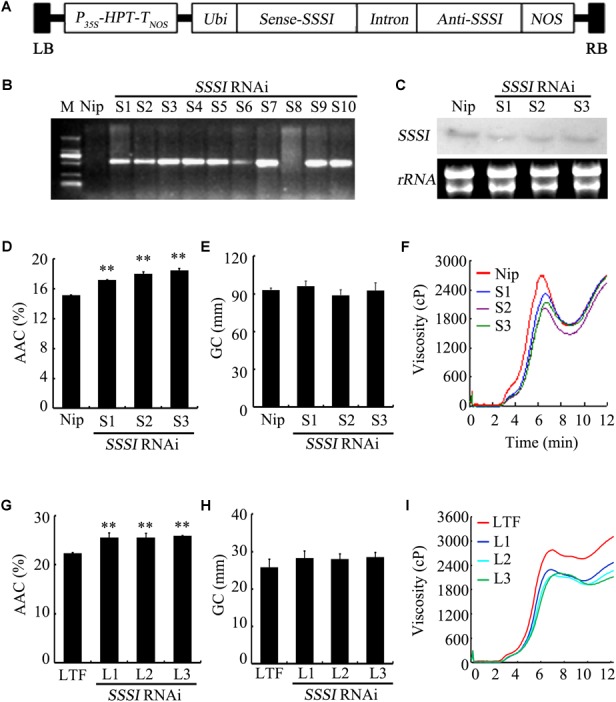
Generation and physicochemical analysis of *SSSI*-RNAi transgenic rice in both *japonica* and *indica* backgrounds. **(A)** An *SSSI*-RNAi construct used for suppression of *SSSI* expression in rice. **(B)** Confirmation of transgenic rice by PCR. **(C)** Expression of *SSSI* analyzed by northern blotting. Apparent Amylose Content (AAC) **(D)**, Gel Consistency (GC) **(E)**, and Rapid Visco Analyzer (RVA) **(F)** analysis of *SSSI*-RNAi transgenic rice in the Nip (*japonica*) background. AAC **(G)**, GC **(H)**, and RVA **(I)** analysis of *SSSI*-RNAi transgenic rice in the LTF (*indica*) background. Asterisks indicate significant differences according to Student’s *t*-tests (^∗∗^*p* < 0.01).

To further characterize the function of *SSSI*, an RNAi strategy was applied to suppress the expression of *SSSI* in both *japonica* cv. Nip and *indica* cv. LTF. A specific *SSSI* DNA fragment was chosen to generate a RNAi cassette that could be used to knock down the expression of *SSSI* in both *japonica* and *indica* (Figure 3A), designated as Nip-*SSSI^j-^*and LTF-*SSS^i-^*. After transgenic rice were obtained, genotyping results indicated that the *SSSI*-RNAi cassette was successfully inserted into the genome of most Nip transgenic lines (Figure 3B). Three representative transgenic lines (S1–S3) were selected for expression analysis, and northern blot assays indicated that transcription of *SSSI* was, indeed, markedly reduced in transgenic rice as expected (Figure 3C). The *SSSI*-RNAi cassette was also successfully transformed into *indica* cv. LTF, and the physicochemical properties of rice were analyzed to evaluate the effect of suppressing *SSSI* expression in both *japonica* and *indica*. In *japonica*, suppression of *SSSI* significantly increased the AAC, but there was no obvious difference in GC (Figures 3D,E).

Rapid viscosity analyzer (RVA) measurements are often used to evaluate rice ECQs, and suppression of *SSSI* led to significant differences in some RVA parameters between transgenic lines and their wild-type (WT) counterparts. For instance, peak viscosity (PKV) and hot paste viscosity (HPV) were decreased in the transgenic lines, suggesting that the ECQs of *SSSI-*RNAi transgenic rice were diminished (Figure 3F). Taking into consideration the changes in physicochemical properties, reduced expression of *SSSI* also led to a similar increase in the AAC but no change in GC in both *indica* and *japonica* (Figures 3G,H). Moreover, the general RVA pattern was identical between transgenic rice and the LTF control. However, almost all primary RVA parameters, including PKV, HPV, and cool paste viscosity (CPV) were markedly reduced (Figure 3I), thus increasing the AAC of both *japonica* and *indica* by knocking down the expression of *SSSI*. Some variation in GC and RVA parameters was evident between the two subspecies.

### Comparison of the Physicochemical and Taste Properties of Rice With Different *SSSI* Alleles

**FIGURE 4:**
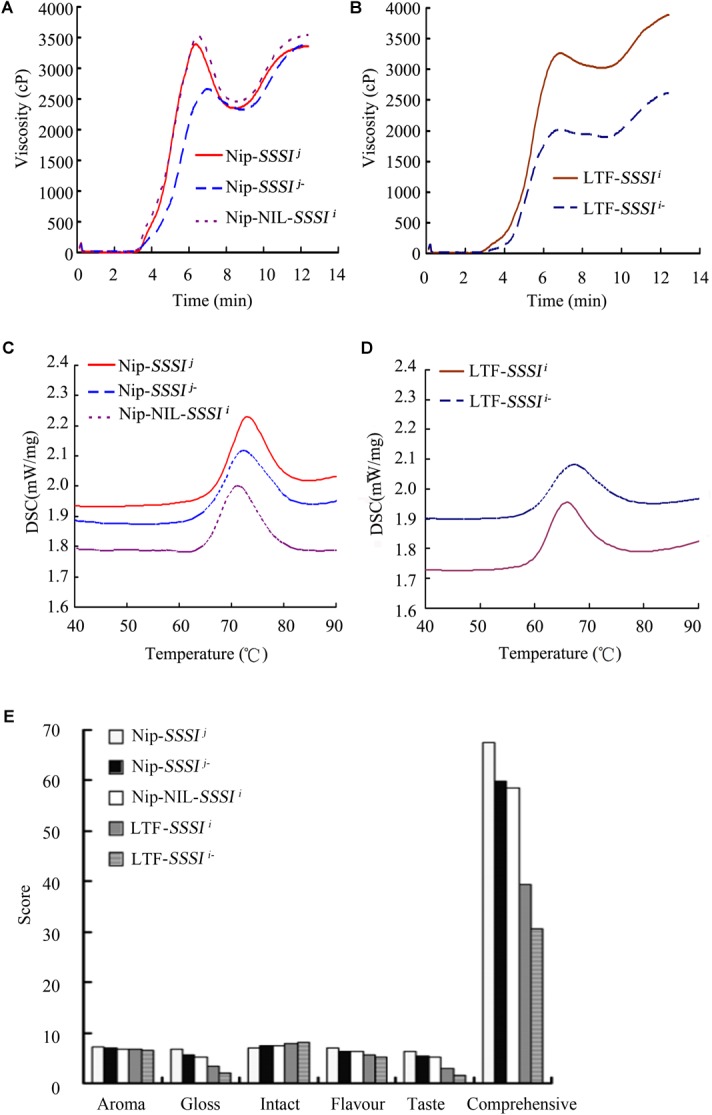
Rapid Visco Analyzer (RVA) and Differential Scanning Calorimeter (DSC) analysis of rice in following suppression of *SSSI* expression or allelic substitution. RVA **(A)** and DSC **(C)** analysis of *SSSI*-RNAi transgenic rice and the NIL-*SSSI^i^* line in the Nip background. RVA **(B)** and DSC **(D)** analysis of *SSSI*-RNAi transgenic rice in the LTF background. The *SSSI*-RNAi transgenic lines in Nip-*SSSI^j^* and LTF-*SSSI^i^* genetic background were designated as Nip-*SSSI^j-^* and LTF-*SSS^i-^*, respectively.

To understand better the influence of *SSSI^j^* and *SSSI^i^* alleles in the same genetic background, the *SSSI^j^* allele in Nip was replaced with *SSSI^i^* to generate the Nip-NIL-*SSSI^i^*. In addition, since the physicochemical analysis of *SSSI* RNAi transgenic rice showed a consistent change among different transgenic lines, a representative transgenic line was selected for analysis and comparison. In general, suppression of *SSSI* significantly altered the RVA curve pattern in both *japonica* and *indica* backgrounds. By contrast, the RVA curves for Nip-*SSSI^j^* and Nip-NIL-*SSSI^i^* were similar, but quite different from that of LTF-*SSSI^i^* (Figures 4A,B). These results suggest that notable differences in the RVA curves of *japonica* and *indica* were partly the result of different *SSSI^j^* and *SSSI^i^* alleles and also due to their genetic interactions with other factors in the genomes of these cultivars. Then, the thermal properties of starch from plants with different *SSSI* alleles were analyzed by DSC. In the *japonica* background, both Nip-*SSSI^j-^* and Nip-NIL-*SSSI^i^* showed similar changes in DSC parameters, including decreased To, Tp, and ΔH (Figure 4C). Furthermore, similar decreases in the same parameters were also observed in the *indica* background (Figure 4D and Table 2).

**Table 2:** Differential scanning calorimetry (DSC) analysis of starch from *SSSI*-RNAi transgenic rice, Nip-NIL-*SSSI^i^* and wild-type controls.

Sample	To (°C)	Tp (°C)	Tc (°C)	ΔH (JG^-1^)
NIP-*SSSI^j^*	67.2 ± 0.71	73.2 ± 0.14	80.55 ± 0.07	12.68 ± 0.18
NIP-*SSSI^j-^*	65.25 ± 0.35^∗∗^	72.15 ± 0.21^∗^	81.55 ± 0.21^∗^	12.14 ± 0.02
NIP-NIL-*SSSI^i^*	64.85 ± 0.21^∗∗^	71.2 ± 0.00^∗∗^	79.2 ± 0.00^∗∗^	12.43 ± 0.02
LTP-*SSSI^i^*	64.5 ± 0.28	65.85 ± 0.07	76.55 ± 0.07	10.20 ± 0.00
LTP-*SSSI^i-^*	60.25 ± 0.67^∗∗^	67.2 ± 0.14^∗∗^	76.15 ± 0.35	9.48 ± 0.21

*To, onset temperature; Tp, peak temperature; Tc, conclusion temperature; ΔH, enthalpy of gelatinization. ^∗^*p* < 0.05; ^∗∗^*p* < 0.01*.

Rice Taste Analyzer is an automatic system for measuring the quality of milled rice and assessing the taste value of cooked rice. The results of TVA showed that the overall taste value of *japonica*-derived rice was notably higher than that of *indica*-derived rice (Figure 4E), which might be due to differences in glossiness and taste between the two rice varieties. Knocking down *SSSI* expression in either *japonica* or *indica* cultivars significantly reduced their comprehensive taste value. Interestingly, the introduction of the *indica* *SSSI* allele into *japonica* cv. Nip resulted in a similar lower taste value to that measured for Nip-*SSSI^j^* rice (Figure 4E), consistent with the fact that *japonica* rice tastes better than the *indica* cultivar. Taking all facts into consideration, the evidence suggests that *SSSI*, and especially the *SSSI^j^* allele, contributes to the taste value of rice.

### Comparison of the Chain Length Distribution of Rice With Different *SSSI* Alleles

**FIGURE 5:**
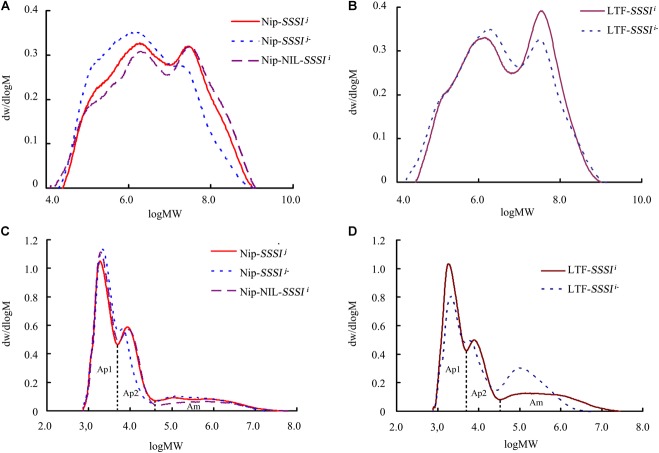
Changes in rice starch chain length distribution following suppression of *SSSI* expression or allelic substitution. GPC analysis of native **(A)** and debranched **(C)** starch from *SSSI*-RNAi transgenic rice and the NIL-*SSSI^i^* line in the Nip background. GPC analysis of native **(B)** and debranched **(D)** starch from *SSSI*-RNAi transgenic rice in the LTF background.

Changes in starch pasting properties, especially peak viscosity, could be due to changes in starch structure. To clarify the influence of *SSSI* on amylopectin structure, the above *SSSI*-RNAi transgenic lines and NILs were used for gel permeation chromatography (GPC) analysis to determine how *SSSI* affects the chain length distribution of amylopectin. Both native and debranched starch from rice with different *SSSI* alleles were used for analysis. Native starch consisted of two fractions, low-MW amylose and high-MW amylopectin (Figures 5A,B). Interestingly, the amylose fraction of starch from *SSSI*-RNAi lines contained a much greater proportion of short chains than that of WT control in both *japonica* and *indica*. By contrast, the amylopectin fraction in the starch from *SSSI*-RNAi lines was greatly reduced when compared with that in WT plants. Subsequently, extracted starch was completely debranched and separated into three major fractions. Ap1 and Ap2 fractions comprise amylopectin; Ap1 mainly contains low MW molecules such as A and short B chains (A + B1 chains) whereas Ap2 is usually composed of long B chains, including high MW molecules. The Ap1:Ap2 ratio generally reflects the extent of amylopectin branching, and the higher the ratio, the greater the branching (Zhang et al., 2016). The third fraction (Am) represents amylose, which consists of a wide range of amylose molecules of different chain lengths (Li et al., 2015). The GPC results revealed that a higher proportion of short chain (Ap1) and lower proportion of long chain (Ap2) molecules of the *japonica* genetic background were present when the expression of *SSSI* was suppressed while the Am fraction was slightly increased when compared with the Nip control (Figure 5C). In NIP-NIL-*SSSI^i^*, the Ap2 fraction was comparable to that in the Nip control, but the Ap1 fraction was much larger and the Am smaller. In the *indica* genetic background, suppression of the expression of *SSSI* significantly decreased the proportion of the Ap1 fraction, and slightly decreased the proportion of the Ap2 fraction, while the Am fraction was significantly increased when compared with the WT control (Figure 5D). The above starch chain length distribution results suggest that *SSSI*-regulated chain length distribution of starch differs in the two subspecies, which might be due to the different *SSSI* allele types and the distinctive genetic background. Thus, modification of the allele type or expression of *SSSI* could modulate the fine structure of starch and subsequently influence the physicochemical properties and ECQs of rice.

### Crystalline Starch Structure

**FIGURE 6:**
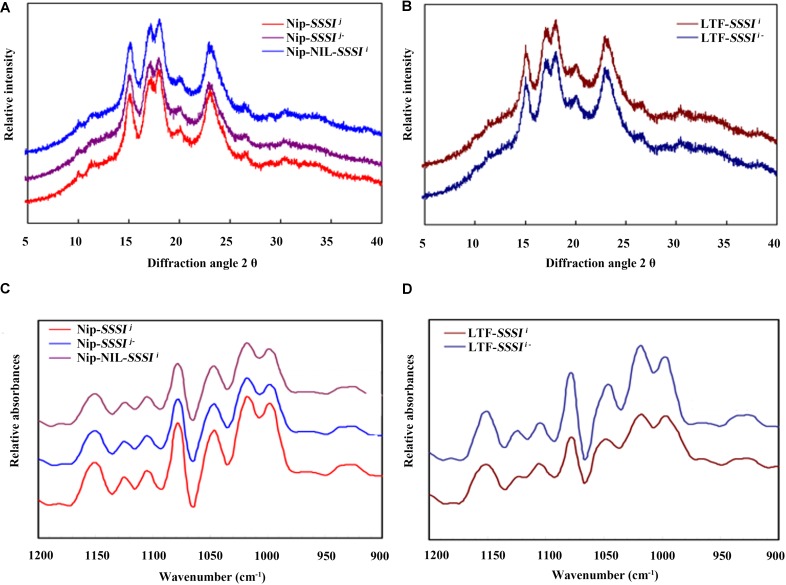
Changes in rice starch crystallinity following suppression of *SSSI* expression or allelic substitution. X-ray Powder Diffraction (XRD) **(A)** and Attenuated Total Reflectance-Fourier Transform Infrared (ATR-FTIR) **(C)** analysis of *SSSI*-RNAi transgenic rice and the NIL-*SSSI^i^* line in the Nip background. XRD **(B)** and ATR-FTIR **(D)** analysis of SSSI-RNAi transgenic rice in the LTF background.

X-ray diffraction is a popular method for structural characterization of crystals. Rice starch exhibits a typical A-type diffraction pattern, with strong diffraction peaks at ∼15° and 23° 2𝜃 and an unresolved doublet at ∼17° and 18° 2𝜃. Our XRD results revealed no differences in the crystal patterns of either *SSSI*-RNAi transgenic lines or *SSSI* NILs (Figures 6A,B). The relative crystallinity calculated from the XRD patterns indicated that the degree of crystallinity declined in *SSSI RNAi* lines, but the extent of the decrease was dependent on the genetic background. In general, the change in the degree of crystallinity was more pronounced in LTF-*SSSI^j-^* lines than in Nip-*SSSI^j-^* lines (Figures 6A,B). The above results imply that modulating the expression of *SSSI* could alter the crystal structure of starch.

The ATR-FTIR is usually used to study the granule crystallinity of starch and the amorphous regions near the granule surface. Moreover, the FTIR intensity ratio of bands at 1022 and 1045 cm^-1^ is correlated with the characteristics of amorphous and crystalline structures in starch, respectively, and a 1045/1022 cm^-1^ ratio is considered a convenient index of ordered starch conformation. Our ATR-FTIR results indicate that suppression of *SSSI* expression significantly reduces the 1045/1022 cm^-1^ ratio (Figures 6C,D and Table 3), suggesting that decreasing *SSSI* expression reduces the crystallinity of starch, thus affecting the physicochemical properties and taste values of rice. This result is consistent with the decreased ΔH values of *SSSI* RNAi transgenic lines in the DSC analysis.

**Table 3:** Relative crystallinity obtained from XRD and absorbance ratios at 1045/1022 cm^-1^ and 1022/995 cm^-1^ obtained from ATR-FTIR.

Sample	Crystallinity (%)	1045/1022 (cm^-1^)	1022/995 (cm^-1^)
NIP-*SSSI^j^*	18.45 ± 0.43	0.728 ± 0.001	3.44 ± 0.002
NIP-*SSSI^j^*^-^	17.44 ± 0.25	0.739 ± 0.002	2.83 ± 0.001^∗∗^
NIP-NIL- *SSSI^i^*	18.00 ± 0.35	0.754 ± 0.001	2.68 ± 0.002^∗∗^
LTF-*SSSI^i^*	15.12 ± 0.63	0.727 ± 0.001	2.39 ± 0.001
LTF-*SSSI^i-^*	14.86 ± 0.42	0.671 ± 0.002	3.71 ± 0.002^∗∗^

*^∗^*p* < 0.05; ^∗∗^*p* < 0.01*.

### Potential Genetic Interaction Between *SSSI* and *Wx*

**FIGURE 7:**
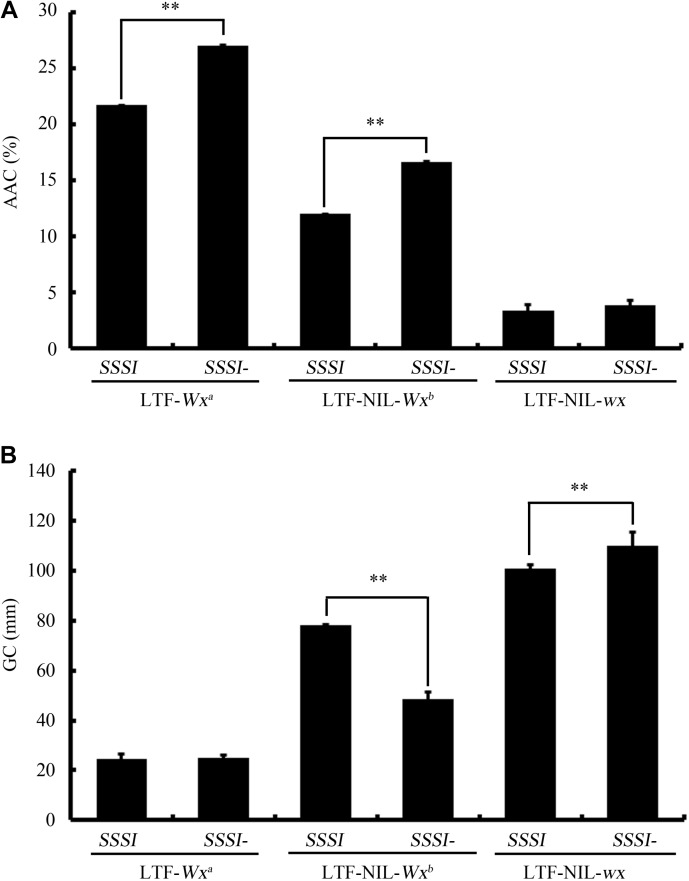
Study the potential genetic interaction of *SSSI* and different alleles of *Wx* gene. The changes of AAC **(A)** and GC **(B)** of rice in response to decreased *SSSI* expression under different *Wx* allele background. Asterisks indicate significant differences according to Student’s *t*-tests (^∗∗^*p* < 0.01).

In spite of the fact that knocking down *SSSI* expression (*SSSI*-RNAi) in both *japonica* and *indica* backgrounds caused consistent changes in most tested physicochemical characteristics, some differences were apparent. One of the major differences between *indica* and *japonica* is the presence of different *Waxy (Wx)* alleles, a major contributor to rice physicochemical properties. To investigate whether *Wx* potentially interacts with *SSSI*, three NILs within the *Wx* locus were generated in the *indica* cv. LTF background, which were designated as LTF-NIL-*Wx^b^*, LTF-NIL-*wx*, and the original LTF-*Wx^a^*. The *SSSI*-RNAi construct was then introduced into these NILs by crossing them. The physiochemical analysis indicated that knocking down *SSSI* significantly increased the rice AAC only in the *Wx^a^* and *Wx^b^* background (Figure 7A). Moreover, changes in GC were quite different among NILs with different *Wx* alleles; GC was decreased in LTF-NIL-*Wx^b^*, increased in LTF-NIL-*wx*, but unchanged in LTF-*Wx^a^* when the expression of *SSSI* was suppressed (Figure 7B). Therefore, the effect of *SSSI* variation on rice quality also depends on its crosstalk with other factors, including the *Wx* gene.

## Discussion

Improving rice grain quality is a major consideration for researchers and rice breeders because rice quality determines market price and consumer demand (Anacleto et al., 2015; Hori et al., 2016). However, as a result of the complicated inheritance, determining rice quality, different consumer preferences, and a lack of effective methodologies for measuring and improving rice quality is far behind improving rice yield (Anacleto et al., 2015). Thus, improving the taste of traditional high-yielding rice is a major challenge in modern rice breeding programs. At present, multiple molecular tools, especially marker-assisted breeding methods, are widely adopted for improving rice ECQs (Lau et al., 2015; Phing et al., 2016). However, progress in breeding of high-quality rice remains limited. In general, starch quality, especially amylose content and amylopectin chain length distribution, is the most important attribute of rice that determines rice ECQs. Therefore, a better understanding of the molecular mechanism of starch quality is essential for rice quality improvement.

The SSSI, together with other SSS family enzymes, functions in amylopectin biosynthesis. It makes the largest contribution to SSS activity in rice endosperm despite consisting of only one isoform. Previous studies indicated that some important starch synthesis-related genes, including *Wx* and *SSSIIa*, have multiple alleles and exert different effects on starch composition and structure in *indica* and *japonica* rice varieties (Tian et al., 2009; Gao et al., 2011; Zhang et al., 2017). Therefore, it is important to explore whether conserved natural variation of *SSSI* contributes to the wide variation in rice starch quality. In this study, we identified four distinct sites of allelic variation in *SSSI*, in both the promoter and exons, with an SNP and an Indel in the promoter and SNPs in exons 6 and 8 (Figure 1A). Correspondingly, four simple PCR-based distinct molecular markers were established. Thus, the four newly established Indel or CAPS markers, together with the previously reported SSR marker 488/489 (Bao et al., 2002), were used to distinguish different *SSSI* haplotypes. Eight genotypes were distinguished among 165 germplasms (Table 1). In spite of the fact that two *SSSI*-related molecular markers were previously reported (Bao et al., 2002; Chen and Bao, 2016), the four sites of natural variation in *SSSI* identified in this study, and their corresponding molecular markers, are novel. Based on this information, we revealed that two haplotypes were closely correlated with *japonica* and *indica* subspecies. For instance, the I-1(I/A/C/C/3) haplotype is predominantly present in *japonica*, and thus we designated it as *SSSI^j^*, whereas the II-1(D/G/T/n/1) allele is specific to *indica*, and hence it was named *SSSI^i^*. To further clarify and compare the functions of *SSSI^j^* and *SSSI^i^*, two strategies were employed; suppression of *SSSI* expression in both *japonica* (*SSSI^j^*) and *indica* (*SSSI^i^*) genetic backgrounds by RNAi and substitution of *SSSI^j^* for *SSSI^i^* in the *japonica* background to generate the NIL Nip-NIL-*SSSI^i^*. The results indicate that knockdown of *SSSI* expression led to a number of consistent changes in physicochemical properties in both *japonica* and *indica*, including increased AC and decreased PKV, To, and ΔH (Figures 4C,D and Table 2). However, there were also some subspecies-specific changes in response to decreased *SSSI* expression. For example, a more prominent decline in HPV and CPV was observed in LTF-*SSSI^i-^* than in Nip-*SSSI^j-^* (Figures 3F,I). Furthermore, changes in the MW distribution of starch were also different in the two subspecies (Figures 5A–D). Previous report indicated that SSSI activity of *indica* rice variety was lower than that of a *japonica* rice variety, thus resulting in the decrease of short chains in the amylopectin of *indica* rice by analyzing F3 endosperms derived from a cross between the two varieties (Takemoto-Kuno et al., 2006). This resulting decrease of short chains is consistent with our result in LTF genetic background. Furthermore, analysis of Nip-NIL-*SSSI^i^* further confirmed that the two major *SSSI* allele types result in differences in starch thermal properties, MW distribution and crystallinity, and subsequently taste values, representing a possible molecular mechanism by which *japonica* generally tastes better than *indica* rice. This knowledge of differences in performance of *SSSI^j^* and *SSSI^i^* alleles in rice, in combination with our newly developed molecular markers, will greatly favor future molecular marker-assisted selection (MAS) of specific *SSSI* allele types in high-quality rice breeding programs.

Experiments were performed to further characterize the biological functions of allelic variation of *SSSI*, especially the Indel in the *SSSI* promoter. Based on the Indel marker, the selected rice varieties were classed into two subgroups, Class-I and Class-II. The majority of rice varieties in Class-I were *japonica*, whereas most varieties belonging to Class-II were *indica*. Several representatives of *japonica* and *indica* rice varieties were selected for the analysis of the *SSSI* expression. In general, *SSSI* was expressed more highly in *indica* than in *japonica* (Figure 1B). Since the promoter is a critical *cis*-element acting in the regulation of target gene expression, two different lengths of *SSSI* promoters were cloned to mimic the *japonica* and *indica* promoters, and their expression patterns were compared to investigate the function of the identified Indel marker. On the whole, the activity of the *GUS* reporter gene driven by the short *SSSI* (0.76 kb) promoter was much higher than that driven by the long (2.1 kb) promoter (Figure 2B). The GUS activity result is consistent with the qRT-PCR result, suggesting that *SSSI* expression was higher in *indica*. Therefore, Indel variation in the promoter could lead to differences in the expression of the *SSSI* gene, which could at least partially account for differences in the performance of *SSSI^j^* and *SSSI^i^* alleles.

Notably, modulation of the structure or expression of a specific starch biosynthetic enzyme is often accompanied by pleiotropic effects on other related enzymes. For instance, suppression of the expression of rice isoforms *SBEI* and *SBEIIb* also results in a large decrease in the activity of SSSI even though accumulation of neither mRNA nor protein were altered (Wang et al., 2018). In maize, the function of SSSIII in normal starch crystallization and prevention of phytoglycogen accumulation is dependent on the existence of only the ISA1 homomer or both the ISA1 homomer and the ISA1/ISA2 heteromer, which illustrates the functional interactions between SSSIII and ISA family enzymes (Lin et al., 2012). In addition, some starch biosynthetic enzymes can directly or indirectly interact with each other to form a functional protein complex in order to regulate starch biosynthesis in a more coordinated manner (Tetlow et al., 2004, 2008; Hennen-Bierwagen et al., 2009; Ahmed et al., 2015; Crofts et al., 2015, 2017). For example, SSSI, SSSIIa, and SSSIIIa, three SSS isozymes, can form an enzymatically active protein complex in rice seeds (Crofts et al., 2015). In maize and wheat, specific SSS polypeptides and SBE enzymes can physically associate with each other to form different multisubunit complexes (Hennen-Bierwagen et al., 2008; Tetlow et al., 2008). In barley, protein-protein interactions among SSSs, SBEs, and starch phosphorylase (SP) led to the formation of different heteromeric enzyme complexes that synthesize A and B granules (Ahmed et al., 2015). In addition to the interplay among enzymes involved in amylopectin biosynthesis, it is important to explore the potential interactions between these amylopectin synthesis-related enzymes and GBSSI, the dominant enzyme responsible for amylose biosynthesis in rice endosperm. Fujita et al. (2011) reported that GBSSI protein abundance in the *SSS3a* mutant was notably higher than that in the WT control (Fujita et al., 2011). Recently, the effects of *Wx* and its interaction with *SSSIII-2* on rice ECQs was investigated using a hybrid combination (Yang et al., 2018). The authors found that differences in the effects of different *SSSIII-2* alleles could be detected only in the *Wx^b^* background and not in the *Wx^a^* background. However, the potential genetic interaction between *Wx* and *SSSI*, the largest component of total SSS activity, has not been systemically studied. Thus, in the present work, we generated two NILs of *Wx* alleles in the LTF (*Wx^a^*) background, designated as LTF-NIL-*Wx^b^* and LTF-NIL-*wx*, which provided valuable genetic material for investigating the interaction between specific factors and different *Wx* alleles. Physiochemical analysis indicated that the effects of *SSSI* on rice AAC and GC are dependent on the *Wx* allele type. For example, knockdown of *SSSI* expression could only significantly increase rice AAC in *Wx^a^* and *Wx^b^* background. More interestingly, a reduction in the expression of *SSSI* led to three distinct GC changes, corresponding to different *Wx* allele types. Specifically, GC was significantly decreased in LTF-NIL-*Wx^b^*, increased in LTF-NIL-*wx*, but remarkably unchanged in LTF-*Wx^a^*. Thus, the effect of *SSSI* variation on rice quality is at least partially dependent on crosstalk with other factors, especially the *Wx* gene. Therefore, it is essential to further explore the potential interactions of other gene pairs and evaluate their effects, thereby expanding the molecular regulatory network related to starch quality and facilitating high-quality rice breeding programs.

## Author Contributions

QQL conceived the research. QFL, XL, and QQL designed the experiments. QFL, XL, CZ, LJ, MJ, MZ, and XF performed all the experiments. QFL, XL, MG, and QQL analyzed the data. QFL and XL wrote the manuscript. All authors read and approved the final manuscript.

## Conflict of Interest Statement

The authors declare that the research was conducted in the absence of any commercial or financial relationships that could be construed as a potential conflict of interest.
