# Supplementary material for: Rice Soluble Starch Synthase I: Allelic Variation, Expression, Function, and Interaction With Waxy
Source: Front Plant Sci. 2018 Nov 13;9:1591. doi: 10.3389/fpls.2018.01591 (PMC6243471; doi:10.3389/fpls.2018.01591)
Supplement: Supplementary file 1 [file Table_1.DOCX]

**SUPPLEMENTARY MATERIAL**

**Rice soluble starch synthase I:** **allelic variation, expression, function and interaction with *Waxy***

Qianfeng Li ^1,2^ ^†^, Xinyan Liu ^1^,^†^ Changquan Zhang^1, 2^, Li Jiang^1^, Meiyan Jiang^1^, Min Zhong^1^, Xiaolei Fan^1^, Minghong Gu^1^, Qiaoquan Liu^1,2*^

^1^ Key Laboratory of Plant Functional Genomics of the Ministry of Education / Key Laboratory of Crop Genetics and Physiology of Jiangsu Province / Jiangsu Key Laboratory of Crop Genomics and Molecular Breeding, College of Agriculture, Yangzhou University, Yangzhou 225009, China

^2^ Co-Innovation Center for Modern Production Technology of Grain Crops of Jiangsu Province / Joint International Research Laboratory of Agriculture and Agri-Product Safety of the Ministry of Education, Yangzhou University, Yangzhou 225009, China


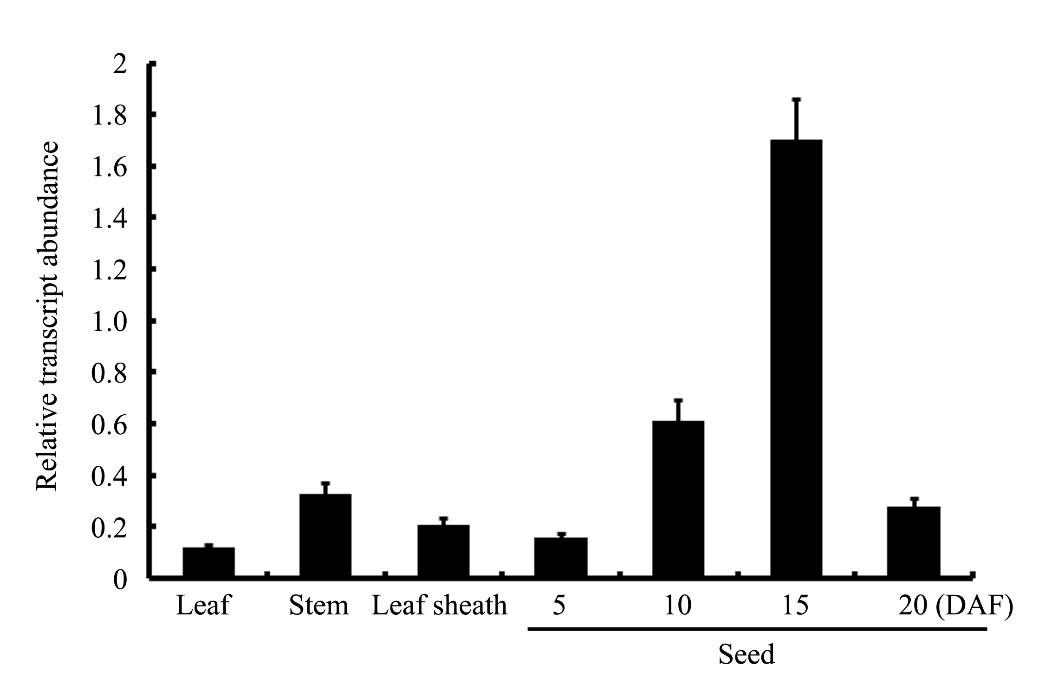


**Supplementary FIGURE 1** Expression profiling of *SSSI* in different tissues and developing seeds detected by real-time RT-PCR. The *Actin* gene was used as an internal control for normalization of gene expression.


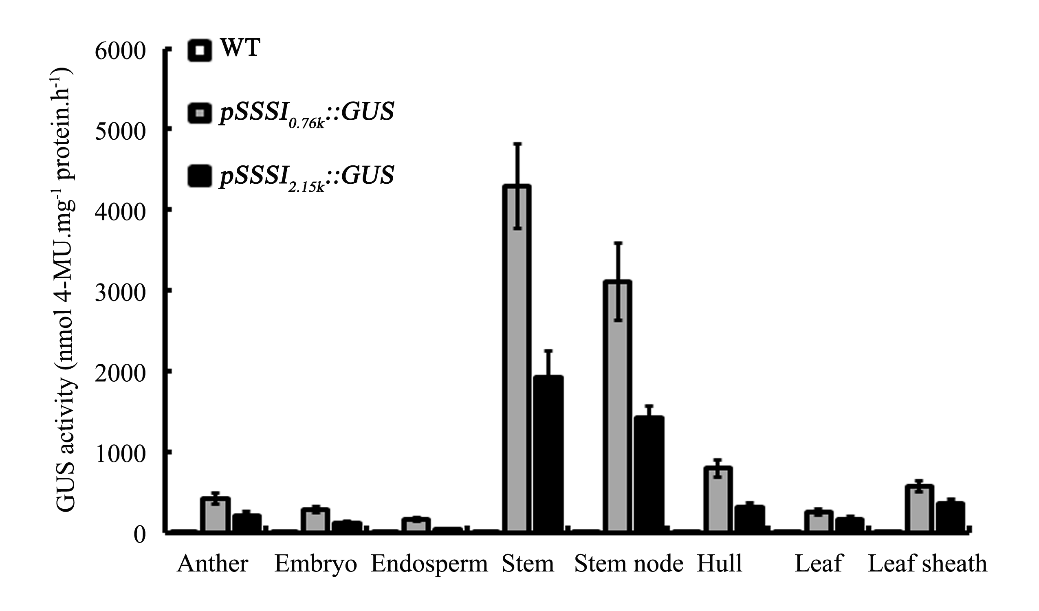


**Supplementary FIGURE 2** GUS activity in different tissues of *pSSSI::GUS* transgenic rice. WT indicates the untransformed wild-type plant. The seed samples analyzed here were collected at 10 DAF. Error bars represent the standard error.

**Supplementary TABLE 1** The primer sequences used in this study

| Primer Names | Sequence ( 5' → 3' ) | Usage |
| --- | --- | --- |
| S001-F | GTAGGCAAGCTGCTACTTGT | For identification of the InDel marker |
| S001-R | CTTGAGGCGCTAATCAGGTT |  |
| 488 | GATCCGTTTTTGCTGTGCCC | For identification of the (AAC) repeat SSR marker |
| 489 | CCTCCTCTCCGCCGATCCTG |  |
| SSSI-13 | ATGGTGTTTACAGGGATGCC | For identification of the SNP in exon 6 |
| SSSI-14 | TTTGACTGTGGCCGTAAGC |  |
| SSSI-20 | CACAGACAAGTTTCTCCCTGAT | For identification of the SNP in exon 8 |
| SSSI-21 | CTTATAGGTAAACCCAGCTC |  |
| SSSI-22 | CCAAAGCCTGTAATAATAAG | For identification of the C/A SNP in SSSI promoter |
| SSSI-23 | CACGCTAAACGAAGAAAT |  |
| SSSIpro-F | CAAGCTTCTCTCTCAACTCTTGCATGGC | For cloning of the 2.15kb SSSI promoter |
| SSSIpro-F1 | GTGAATTCCATACGGAAACCTTACG | For cloning of the 0.76kb SSSI promoter |
| SSSIpro-R | TCCATGGTCGCCCCGAGGAACCT | For cloning of the SSSI promoter |
| SSSI-10 | AACTAGTCTCCAGCGGGTGCGGAG | For generation of the SSSI RNAi construct |
| SSSI-11 | TGGATCCAGGTCTATGATATGAGGGATGATC |  |
| SSSIqRT-F | GGGCCTTCATGGATCAACC | For qRT-PCR analysis of SSSI expression |
| SSSIqRT-R | CCGCTTCAAGCATCCTCATC |  |
| ActinqRT-F | CCAAGGCCAATCGTGAGAAGA | As reference gene for the qRT-PCR analysis |
| ActinqRT-R | AATCAGTGAGATCACGCCCAG |  |
| A224 | TTGTGGCTAGTGAGCAGG | For generation of the SSSI probe for Northern blot assay |
| A225 | GACTGGCACAAGACTGGC |  |
| HP1 | GCTGTTATGCGGCCATTGTC | For genotyping SSSI RNAi transgenic rice |
| HP2 | GACGTCTGTCGAGAAGTTTC |  |
